# Transporting Hydrogel via Chinese Acupuncture Needles for Lesion Positioning Therapy

**DOI:** 10.1002/advs.202200079

**Published:** 2022-04-11

**Authors:** Feng Lin, Zhen Wang, Lei Xiang, Longxi Wu, Yupu Liu, Xiaobing Xi, Lianfu Deng, Wenguo Cui

**Affiliations:** ^1^ Department of Orthopaedics Shanghai Key Laboratory for Prevention and Treatment of Bone and Joint Diseases Shanghai Institute of Traumatology and Orthopaedics Ruijin Hospital Shanghai Jiao Tong University School of Medicine 197 Ruijin 2nd Road Shanghai 200025 P. R. China

**Keywords:** Chinese acupuncture, hydrogel, lesion positioning therapy, microneedle, osteoarthritis

## Abstract

Lesion positioning therapy optimizes medical treatment by directly targeting lesions. However, strong physical barriers greatly hinder its wide use. Here, the Chinese acupuncture needles (CA‐needles) with a screw‐thread structure at the tip (ST‐needle) and the hydrogel with the function of adhesive metal and loaded drug sustained‐release structure are designed, through the minimally invasive and precise positioning of lesions by ST‐needles, the dry‐wet conversion of hydrogel with absorbing fluids and swelling, and the rotation back of ST‐needles, the hydrogel is precisely positioned in the subchondral bone with physical barrier to achieve precise positioning therapy for lesions. In vitro experiments show that the ST‐needle penetrates the physical barrier of cartilage and enters the subchondral bone. Simultaneously, the hydrogel transfer efficiency of the ST‐needle (73.25%) is significantly higher than that of the CA‐needle (29.92%) due to the protective effect of the screw‐thread structure. In vivo experiments demonstrate that precise positioning in subchondral bone in osteoarthritis rats with ST‐needles effectively inhibits abnormal subchondral bone remodeling, alleviating the degeneration and degradation of cartilage. Therefore, ST‐needles achieve lesion positioning therapy through minimally invasive penetration of physical barriers, precisely positioning within lesions, and delivering hydrogel to release drugs.

## Graphical abstract

In order to expand the application of lesion positioning therapy, screw‐thread tipped Chinese acupuncture Needles (ST‐needle) are designed to penetrate physical barriers like cartilage in a minimally invasive manner to accurately deliver drug‐loaded hydrogel to target lesions, and release the drug over a sustained period. Therefore, the ST‐needle system is a promising method for lesion positioning therapy.

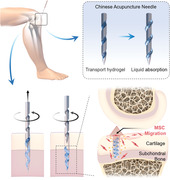

## 1 Introduction

Lesion positioning therapy includes optimal disease control strategies and iatrogenic damage control strategies, which play an important role in medical treatment. The advantage of lesion positioning therapy is that it can precisely localize drugs to the lesion and produce an enrichment effect, which can greatly improve drug efficacy and significantly reduce the side effects caused by treatment.^[^ ^1^, ^2^, ^3^, ^4^ ^]^ At present, lesion positioning treatment of various diseases has been achieved through the application of functional biological materials, such as hydrogel microspheres^[^ ^5^ ^]^ and electrospinning.^[^ ^6^ ^]^ However, the physical barrier of the physiological structure of some tissues or organs (such as intervertebral discs^[^ ^7^ ^]^ and cartilage^[^ ^8^ ^]^) greatly hinders the implementation of lesion positioning therapy. Therefore, designing a drug‐delivery system that can powerfully penetrate the physical barriers of various tissues and organs and achieve accurate localization within lesions is a major problem in urgent need of a solution. Currently, Chinese acupuncture (CA) is the most mature lesion positioning therapy in traditional Chinese medicine and has been clinically applied for thousands of years. Its efficacy has been shown over time, and its use is popular worldwide.^[^ ^9^, ^10^, ^11^, ^12^ ^]^ A microneedle with a tip diameter of less than 200–300 µm and a length of 110 mm is used to penetrate the physical barrier of various tissues and organs, directly contacting the lesion, where it precisely stimulates the lesion and changes its microenvironment to alleviate or even treat the disease. Moreover, the micron size of Chinese acupuncture needles (CA‐needles) makes them minimally invasive, and patients feel almost no pain during acupuncture treatment, with almost no bleeding at the acupuncture site. After acupuncture treatment, the micron trauma left by the needle heals quickly, and patients can immediately leave the treatment room and resume normal life without bandaging or other medical treatment. Therefore, in China, patients have a high acceptance of acupuncture and moxibustion, and there has been no psychological rejection of the technique in the face of invasive therapies. Patients generally accept the frequency of acupuncture treatment at three to five times per week, which also ensures its significant efficacy.^[^ ^13^, ^14^, ^15^ ^]^ However, presently, acupuncture therapy mainly uses physical stimulation to intervene and treat the lesion site. Facing different diseases, different pathological microenvironments often lack pertinence, which seriously limits the further expansion of acupuncture and moxibustion in the field of lesion positioning therapy. Therefore, further development of CA could ensure lesion positioning treatment according to the microenvironment of different diseases.

Microneedle patches have been widely used in the medical field due to their functional design, enabling lesion positioning therapy for various disease microenvironments. Presently, microneedle patches have achieved precise localization of drug release for the treatment of various solid tumors^[^ ^16^ ^]^ and organs.^[^ ^17^ ^]^ Although microneedle patches effectively penetrate a variety of tissues and organs and deliver drugs to specific sites, they are an ideal biomaterial for lesion positioning therapy. However, existing microneedle materials have two defects. First, the penetration depth is not sufficient, and they can only target shallow tissues. Second, they lack the hardness to penetrate the tough physical barriers of some tissues and organs (such as cartilage and ligaments). These two deficits exist because the materials chosen to make microneedles are mainly organic polymers and silicon‐based materials,^[^ ^18^ ^]^ seriously hindering the application and expansion of microneedles in the field of lesion positioning therapy. Therefore, Chinese acupuncture needles (CA‐needles) are proposed as the microneedle material for lesion positioning therapy. CA‐needles are made from metals and are physically hard, and cannot be matched by organic polymers and silicon‐based materials. CA‐needles can be made hundreds of millimeters long, lengths that can easily reach deep tissues of the human body and can penetrate physical barriers of hard tissues through entry at the skin. At present, acupuncture has been widely used to treat diseases of the muscles,^[^ ^19^ ^]^ intervertebral discs,^[^ ^20^ ^]^ sciatic nerves,^[^ ^21^ ^]^ and other tissues deep in the body. However, targeted treatment with CA‐needles based on metal materials is difficult to achieve due to their single chemical composition and lack of chemical groups that can be used for chemical modification. However, hydrogels have great potential for the functionalization^[^ ^22^ ^]^ of CA‐needles. Therefore, designing a CA‐needle that can load a hydrogel and deliver it in a precise and minimally invasive manner is a major problem that urgently needs to be solved.

Osteoarthritis (OA) is the most common chronic degenerative joint disease worldwide.^[^ ^23^ ^]^ Many functional biomaterials have been developed for lesion positioning treatment of OA. As previously reported, Yang et al.^[^ ^24^ ^]^ designed an efficient liposome (Lipo)‐anchoring platform to combine Lipo with a photo‐crosslinked gel matrix that continually releases drugs into the cartilage in the joint cavity. Lei et al.^[^ ^25^ ^]^ designed another injectable hydrogel microsphere that recruits stem cells. Thus, the cartilage defect can be repaired in the articular cavity. These biomaterials can achieve positioning therapy of knee cartilage, effectively improving the injury and degeneration of the cartilage. However, in subchondral bone disease,^[^ ^26^, ^27^, ^28^, ^29^ ^]^ an early development in OA, almost no biomaterials can penetrate the physical barrier of cartilage to precisely localize treatment for subchondral bone. Current studies have found that abnormal secretion of the subchondral bone cytokine transforming growth factor‐*β*1 (TGF‐*β*1)^[^ ^30^ ^]^ in OA leads to subchondral bone disease (including abnormal remodeling, sclerosis, and abnormal mineralization),^[^ ^28^, ^31^, ^32^ ^]^ and ultimately accelerates the progression of OA. However, regulating the abnormal secretion of TGF‐*β*1 by systemic administration leads to side effects. Meanwhile, subchondral bone stem cells are a key factor in repairing cartilage damage. In clinical practice, drilling is often performed at the cartilage‐damaged site during knee surgery to promote the migration of subchondral bone stem cells to the cartilage defect to repair the cartilage.^[^ ^33^ ^]^ However, this treatment can only be done in patients undergoing surgery. Lesion positioning therapy for subchondral bone can improve the mechanical environment of the cartilage and alleviate cartilage damage from the root, and mesenchymal stem cells (MSCs) in the subchondral bone also play an important role in repairing cartilage damage. Therefore, we investigated the use of CA‐needles to penetrate the physical cartilage barrier, to deliver a carrier hydrogel to the subchondral bone to regulate the abnormal secretion of TGF‐*β*1, and to construct a migration channel for subchondral bone stem cells to achieve lesion positioning treatment for subchondral bone in a minimally invasive manner.

**Scheme 1:**
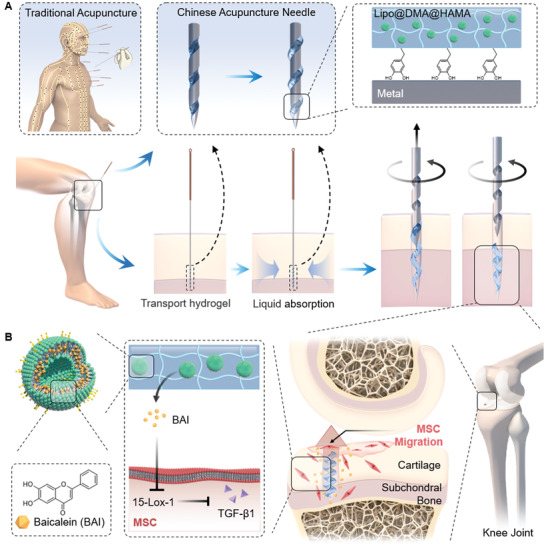
Schematic diagram of lesion positioning therapy. A) Chinese acupuncture needles (CA‐needles) with a screw‐thread structure at the tip (ST‐needles) were designed to penetrate the subchondral bone and transport the hydrogel. B) The pores left by the ST‐needles promoted MSC migration, and the hydrogel continuously released BAI to regulate cytokine secretion.

However, in the process of lesion positioning therapy with CA‐needles loaded with hydrogel penetrating into subchondral bone, a major problem is that the hydrogel does not detach from the surface of the CA‐needle to be successfully delivered to the lesion site. In this study, we designed a CA‐needle with a screw‐thread structure at the tip (ST‐needle) (**Scheme** **1**). The ST‐needle and hydrogel constituted the ST‐needle drug delivery system, which for the first time realized the breakthrough of the strong physical obstacles of cartilage and cortical plate, and precisely located the drug in the subchondral bone. This was something no previous drug delivery system had been able to do. Briefly, A screw‐thread structure loaded hydrogel was realized through the polymer interface interaction, and the screw‐thread groove structure and the dry‐wet transformation of the hydrogel were used to successfully transport the drug‐loaded hydrogel into the lesion and continuously release the drug. First, we used the photo‐crosslinking reaction between N‐[2‐(3,4‐Dihydroxyphenyl)ethyl]‐2‐methylprop‐2‐enamide (DMA) and hyaluronic acid (HA) methacrylate (HAMA) to prepare a DMA@HAMA hydrogel with an adhesive polymer interface. Subsequently, we selected Lipo as the carrier of Baicalein (BAI) to achieve the sustained release of the drug and embedded the Lipo into the DMA@HAMA hydrogel to prepare a Lipo@DMA@HAMA hydrogel that can adhere to the ST‐needle. Finally, Lipo@DMA@HAMA was loaded into the thread groove of the ST‐needle and was air‐dried. Due to the interaction between the adhesive polymer interface of the hydrogel and the metal and the protective effect of the thread groove on the hydrogel, the hydrogel will not fall off when the tissue or organ is punctured by the ST‐needle. When the ST‐needle enters the lesion, the hydrogel swells in contact with body fluids, squeezing and adhering to the surrounding tissues. As the ST‐needle is rotated and withdrawn, the hydrogel is left at the site of the lesion, and the drug is continuously released to achieve lesion positioning therapy. To verify that our design is correct and effective, we would conduct a series of tests on the ST‐needle system. First, we tested the controlled release performance, swelling degradation performance, and metal adhesion performance of the hydrogel, so as to prove that the hydrogel designed by us could be successfully combined with ST‐needle and could stay in the subchondral bone for sustained controlled release of drugs. Second, we would test the ability of ST‐needle to penetrate cartilage to prove that our designed ST‐needle could easily penetrate into subchondral bone in a variety of articular cartilage. Third, in in vitro experiments, we strictly proved that ST‐needle could successfully deliver hydrogel to subchondral bone through fluorescence labeling experiment and histologic section. Finally, in animal experiments, we verified the efficacy of the ST‐needle system, which was significantly better than the joint cavity injection. Therefore, the ST‐needle effectively penetrates the physical barrier of the cartilage, accurately locating and reaching the subchondral bone, and can successfully deliver the Lipo@DMA@HAMA to the subchondral bone lesions, inhibiting the apoptosis of the cartilage cells and the degradation of the cartilage matrix, effectively alleviating OA.

## 2 Results and Discussion

### 2.1 Minimally Invasive CA‐Needle and the Design of the ST‐Needle

**Figure 1:**
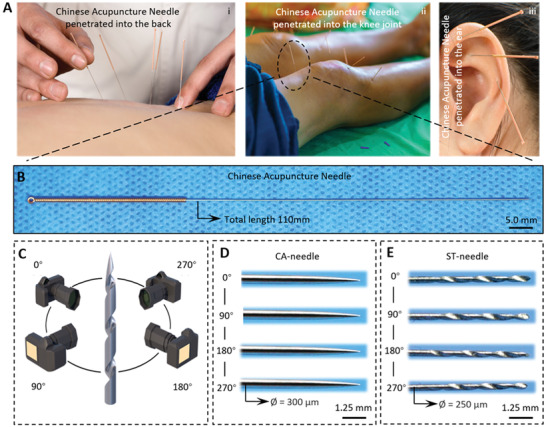
Characteristics of the CA‐needle and ST‐needle. A) Clinical application of CA‐needles. All the photos were purchased from https://www.paixin.com. (i: License ID: 100624901, ii: License ID:100624849, iii: License ID: 100624900). B) Morphological schematic of a CA‐needle. C) The camera was used for 360° macro photography of the microneedle. D) 360° display of the CA‐needle tip. E) 360° display of an ST‐needle.

The CA‐needle, the most mature precision positioning treatment method in traditional Chinese medicine, has been widely used in the treatment of various body parts and diseases, including the back (**Figure** **1**Ai), joints (Figure 1Aii), and face (Figure 1Aiii). The CA‐needle can be used to accurately locate and stimulate lesions. The diameter of the CA‐needle tip is within 200 µm, and its length is ≈10–15 cm. The tail is wrapped with copper wire, which can increase the friction force and help doctors better hold the CA‐needle so it can be easily inserted into the human body or rotated during acupuncture (Figure 1B). Due to the micron size of the CA‐needle, acupuncture treatment is minimally invasive with minimal trauma to patients. In clinical practice, after the removal of acupuncture needles, there is almost no bleeding on the skin, and the needle holes caused by acupuncture cannot be seen by the naked eye. After receiving acupuncture treatment, the patient is able to leave the examination room immediately and resume normal life without additional treatments such as skin dressing.

To further verify the minimally invasive effect of CA‐needles on tissues or organs in vivo, we selected three tissues and organs most prone to massive bleeding after injury: abdominal veins, liver, and spleen. Simultaneously, we selected 1‐mL syringes as the Control group. CA‐needles and syringes were used to pierce the abdominal vein, liver, or spleen to observe the bleeding of various tissues and organs. From the experimental photos, it can be seen that after the CA‐needle was inserted into the abdominal vein of rats, there was only a small amount of bleeding, and almost no blood was found on the gauze underneath. After the syringe was inserted into the abdominal vein of the rat, a large amount of blood immediately poured out of the puncture site, and the gauze underneath was immediately soaked with blood (Figure S1A, Supporting Information). Subsequently, the liver (Figure S1B, Supporting Information) and spleen (Figure S1C, Supporting Information) were also punctured. At 3–5 min after insertion, the liver and spleen of the CA‐needle group showed no obvious blood exudation, and the gauze underneath only adhered to a small amount of blood. The liver and spleen of the Control group showed exudated blood on the surface of the organ, and the gauze underneath also absorbed a lot of blood. This shows that CA‐needles are less invasive than traditional syringes for tissues and organs in the body (Figure S2, Supporting Information).

Because traditional CA‐needles are mostly made of stainless steel, it is not possible to use electron microscopy to microscopically observe acupuncture. Therefore, to better display the tip of the acupuncture needle, we used a macro lens to photograph the tip (Figure 1C). The traditional acupuncture needle has a sharp, smooth tip and no special structure to hold the hydrogel (Figure 1D). To enable traditional acupuncture to load a hydrogel, we designed a screw‐thread structure at the tip of the needle to obtain a screw‐thread microneedle (ST‐needle). We have found a professional metal processing factory to produce ST‐needles. After repeated experiments, we finally chose 314 medical stainless steel as the material of ST‐needle. The diameter of the ST‐needle is 0.25 mm, the pitch is 0.9 mm, and the groove depth is 0.05 mm. Of course, these parameters can be changed according to actual requirements. From the macro photograph, it can be seen that we carved a groove ≈200 µm wide on the tip of the microneedle, which is threaded from tip to tail (Figure 1E).

Then, we envisioned the clinical application of ST‐needle system, and we would carry out our lesion positioning therapy in the “acupuncture treatment room,” because the treatment process for patients is almost the same as traditional acupuncture treatment. Since the ST‐needle is inserted from the “acupoint” of the patient's skin, the patient will feel numb and sour, which is a very comfortable feeling. During the “acupuncture treatment,” the doctor will leave the acupuncture in the body for ≈15–30 min, so that the patient can fully enjoy the comfortable feeling, so as to relieve the pain caused by the disease, and our ST‐needle system, which is based on acupuncture, also stays in the body for that long. This is enough time for the hydrogel to fully swell in the body (we have hydrogel swelling experiments to support this idea). At the same time, in order to facilitate doctors to judge whether hydrogel is detached from the needle when the needle was removed in clinical application scenarios, we plan to produce the ST‐needle with non‐toxic black coating in the future (this is easy to achieve in industry). The tip of the ST‐needles loaded with hydrogel will appear milky white. When the ST‐needles are removed from the patient and the doctor sees that the tip of needle turns black almost as black as the body, doctor can tell that the hydrogel has been completely removed from the tip. The above is only a preliminary idea of the clinical application of ST‐needle system, and the actual use of ST‐needle will definitely face some unexpected problems, which require continuous optimization in practice. In future studies, we will continue to improve the ST‐needle, including the width and depth of the groove, the density of the screw‐thread, and the extension distance of the screw‐thread at the tip, so the ST‐needle can be adapted to different hydrogels with different physical properties and to meet different treatment needs of various diseases.

### 2.2 Synthesis of the Hydrogel and Loading onto ST‐Needles

**Figure 2:**
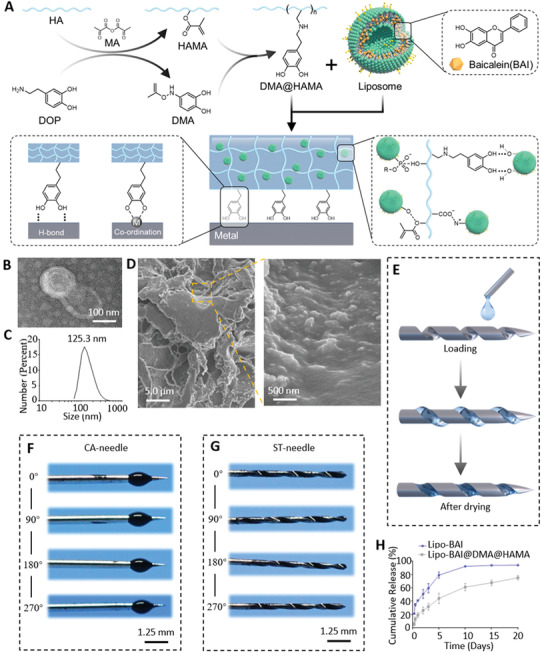
Demonstration of hydrogel‐modified ST‐needle. A) Schematic diagram of hydrogel synthesis with the adhesive metal surface property. B) Transmission electron microscopy micrographs of liposomes (Lipos). C) The particle size distribution of Lipos in aqueous solution. D) Scanning electron microscopy images of the Lipo@DMA@HAMA hydrogel. E) Schematic diagram of the hydrogel‐modified ST‐needle. F) 360° display of the CA‐needle tip with the hydrogel. G) 360° display of the ST‐needle tip with the hydrogel (The hydrogel was dyed dark blue for easy observation). H) In vitro release of BAI from Lipo‐BAI and Lipo‐BAI@DMA@HAMA *(n* = 3).

After the innovative preparation of ST‐needle, we need to endow the ST‐needle with greater modifiable potential. Therefore, we designed the system of ST microneedles and hydrogel. We innovatively designed a hydrogel that can adhere to metal, and inserted drug‐carrying Lipos into the hydrogel to achieve long‐term sustained release of the drug (**Figure** **2**A). Crosslinked Gelatin methacryloyl (GelMA)^[^ ^34^ ^]^ is the most commonly used material to make hydrogels. However, as protein‐based materials, after its degradation in the human body, the free heterologous proteins may have hidden dangers. Hyaluronic acid (HA) is a natural glycosaminoglycan polymer widely present in cartilage matrix and joint fluid, and is widely used in cartilage regeneration due to its good biocompatibility.^[^ ^35^ ^]^ Therefore, HAMA was prepared as hydrogel material by chemical combination of HA and methacryloyl. In addition to the general properties of hydrogel, the HAMA material itself also has a certain protective effect on cartilage, and has been widely used in the treatment of cartilage related diseases.^[^ ^36^ ^]^ Dopamine is an important derivative of dihydroxy phenylalanine and has similar adhesion property to mussels.^[^ ^37^ ^]^ We innovatively modified methacryloyl on dopamine to obtain DMA, and DMA can participate in the photo‐crosslinking reaction of HAMA through methacryloyl, so as to obtain HAMA with dopamine group modification. Finally, we obtained a new HAMA hydrogel with adhesive properties. Subsequently, we prepared drug‐carrying Lipos of uniform size using the thin‐film method (Figure S3, Supporting Information), dispersed liposomes were obtained (Figure 2B), and the diameter of the Lipos was ≈125 nm (Figure 2C). Subsequently, we used DMA and HAMA for photo‐crosslinking to prepare a hydrogel that can adhere to metal surfaces. To prepare the hydrogel containing Lipos, we added Lipos into the mixed solution of DMA and HAMA, and after the photo‐crosslinking reaction, the Lipos were firmly bound to the solidified hydrogel (Figure S4A, Supporting Information). Subsequent experiments confirmed that DMA@HAMA effectively adhered to the metal (Figure S4B, Supporting Information). Lipo@DMA@HAMA was observed via scanning electron microscopy, showing that Lipos nanoparticles with a diameter of ≈100 nm were evenly distributed inside the porous hydrogel (Figure 2D). The above experiments demonstrate that we have successfully prepared a hydrogel that can adhere to a metal surface that was loaded with Lipos, which can release drugs over a long time.

To combine the hydrogel with the ST‐needle, we used a 1 mL syringe to extract an appropriate amount of Lipo@DMA@HAMA aqueous solution, which was dropped onto the CA‐needle tip and the ST‐needle tip and cured by ultraviolet (UV) irradiation for 20 min (Figure 2E). In the same way as described above, we took macro photographs of the CA‐needle tip and the ST‐needle loaded with the hydrogel and dyed the hydrogel dark blue for easy observation. The hydrogel on the tip of the CA‐needle is directly exposed to the metal surface and is significantly higher than the contour of the CA‐needle (Figure 2F). However, on the tip of the ST‐needle, the hydrogel was contained in the groove of the screw‐thread, and the hydrogel was not significantly higher than the contour of the ST‐needle (Figure 2G). Thus, the screw‐thread structure of the ST‐needle can protect its load of hydrogel during the process of puncturing human tissues.

Finally, the hydrogel was used as a biomaterial to load drugs, and its drug release performance was further tested (Figure 2H). The final concentration of the drug was controlled at 1 mg mL^−1^ in the preparation of drug‐carrying Lipos. It was calculated that the liposome contained 70 micrograms of BAI per 1 mg of freeze‐drying Lipos. Subsequently, the drugs were loaded into Lipos (Lipo‐BAI) and Lipo@DMA@HAMA hydrogel composite (Lipo‐BAI@DMA@HAMA). The two biomaterials were placed in a dialysis membrane and immersed in polyphosphate‐buffered saline (PBS) at 37 °C, and the drug content in PBS was detected periodically. The drugs in the Lipos were released fairly quickly, and by day 5, they were ≈80% released. The Lipo@DMA@HAMA composite hydrogel material had a better sustained release effect, and ≈50% of the drug was released on the fifth day. Meanwhile, the drug release lasted for ≈3 weeks. Therefore, the Lipo@DMA@HAMA composite material has an ideal slow‐release performance.

The joint retention time was another important factor that affected the therapeutic efficacy of the hydrogel drug carrier. The ideal hydrogel should be weakly biodegradable. As seen from Figure S5, Supporting Information, In order to better simulate the joint cavity environment, we added collagenase (1 UmL^−1^) into PBS at 37 °C. And the hydrogel was made into small, dispersed spheres to make good contact with the collagenase (Figure S5A,B, Supporting Information). the hydrogel underwent a two‐stage degradation process, in which a rapid degradation occurred in the first 14 days, and then followed by a slow degradation till the hydrogel was completely degraded on day 35 (Figure S5C, Supporting Information). Therefore, the hydrogel prepared by us has the ideal weakly biodegradable efficiency, which not only takes enough time to achieve controlled release of the drug, but also does not affect tissue regeneration at the later stage. Since the hydrogel had a sponge‐like 3D network structure that consisted of hydrophilic polymers groups, they were capable of imbibing large amounts of fluids. In this study, the hydrogel swelled to ≈35 times of its original value after 30 min of incubation in deionized water, then slightly reduced during the following 30 min, and finally reached to ≈30 times after stabilization (Figure S5D, Supporting Information). Therefore, the hydrogel has good swelling properties, which can meet the needs of lesion positioning therapy of ST‐needle system.

### 2.3 The ST‐Needle Penetrates Cartilage and Reaches the Subchondral Bone

**Figure 3:**
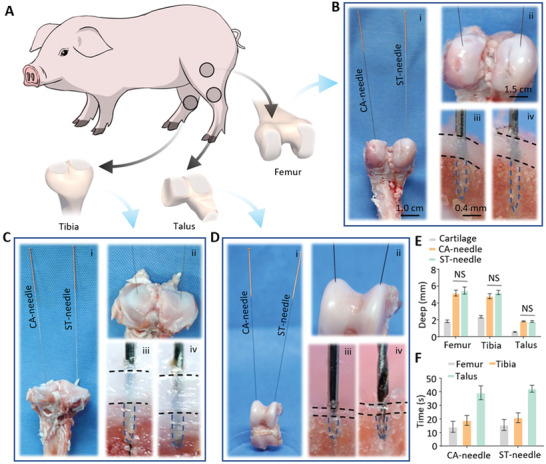
Perforation experiments were performed in porcine articular cartilage using the CA‐needle and the ST‐needle. A) The three main articular cartilages of pigs. B–D) Perforation experiment of articular cartilage of the femur, tibia, and talus: i) Photos of the full display. ii) Photos of the partial display. iii) The CA‐needle inside the cartilage. iv) The ST‐needle inside the cartilage. E) Depth of the CA‐needle and the ST‐needle perforation into cartilage (*n* = 3). F) Time of the CA‐needle and the ST‐needle perforation into cartilage (*n* = 3). (The black dotted line shows the outline of the cartilage and the blue dotted line shows the outline of the CA/ST‐needle; NS: no significance).

We hope to use the ST‐needle system to treat subchondral bone lesions in patients with osteoarthritis. Therefore, we needed to verify the ability of ST microneedles to penetrate cartilage and reach subchondral bone. To demonstrate the ability of ST‐needles to precisely target the subchondral bone, we used pigs as model animals for in vitro experiments. We selected the three most common articular cartilage surfaces seen clinically in joint diseases: the femoral condyle, the tibial plateau, and the talus, to verify that the ST‐needle can be widely applied to the precise treatment of joint diseases at multiple sites (**Figure** **3**A). At the same time, we chose CA‐needles as the Control group to better demonstrate the puncture ability of ST‐needles. We used CA‐needles on the left side of each articular surface and ST‐needles on the right side and recorded the microneedle puncture of the cartilage using macro photography. Simultaneously, after we tested the microneedle puncture into the cartilage, we dissected the punctured site to directly observe whether the microneedle entered the subchondral bone and measured the depth of the microneedle penetration into the subchondral bone (Figure 3B–D). The penetration depth of the two kinds of microneedles in the cartilage of the femur, tibia, and talus exceeded the thickness of the cartilage (Figure 3E). This shows that the CA‐needle can effectively penetrate the cartilage barrier to reach the subchondral bone. Moreover, screw‐thread modification of the microneedle tip did not weaken the ability of the ST‐needle to penetrate the physical barrier. Subsequently, we also statistically analyzed the time of each implementation of acupuncture (Figure 3F). Although the cartilage of the talus is hard, it took more time to penetrate the subchondral bone, but in general, the acupuncture time was less than 1 min, which was also within the allowable clinical practice of acupuncture. In conclusion, the ST‐needle can penetrate cartilage, can precisely localize within subchondral bone, and has a high potential for clinical application.

### 2.4 Ability of ST‐Needle to Transport Hydrogel

**Figure 4:**
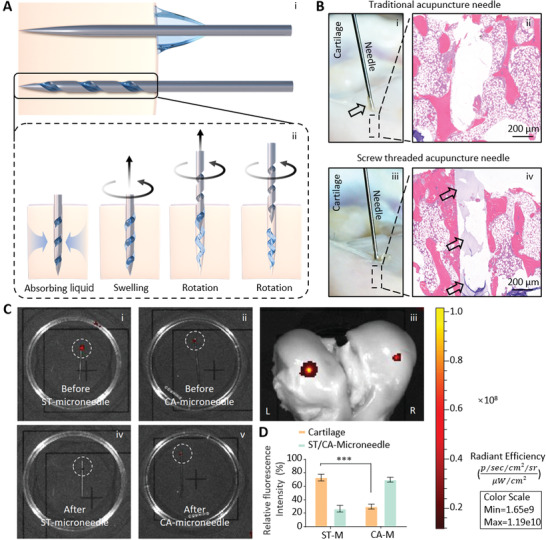
The hydrogel was transported by the ST‐needle. A) Schematic diagram of the ST‐needle transport hydrogel. i) The CA‐needle could not transport the hydrogel into the tissue, while the ST‐needle successfully transported the hydrogel into the tissue. ii) The dried hydrogel absorbed liquid from the tissue, which swelled to make close contact with the surrounding tissue. The ST‐needle rotated and withdrew, leaving the hydrogel in the tissue (The blue arrows represent the fluid absorbed by the hydrogel in the surrounding tissue). B) In vitro experiment verified the effect of ST‐needle transport hydrogel. i) The hydrogel‐loaded CA‐needle was inserted into the pig cartilage (the black arrow indicated hydrogel blocked by cartilage). ii) the histologic section of subchondral bone. iii) The hydrogel‐loaded ST‐needle was inserted into the pig cartilage. iv) The histologic section of subchondral bone (the black arrow indicated hydrogel that had been successfully transported to the subchondral bone). C) Cy‐7‐labeled hydrogel was used to verify that the ST‐needle could transport more hydrogel into the cartilage than the CA‐needle. D) Quantitative analysis of fluorescence intensity (*n* = 3). (*** *P* < 0.001).

The key of ST‐needle system to complete lesion positioning therapy is to accurately transport functional hydrogel into the lesion. Therefore, we investigated the ability of the ST‐needle to deliver a hydrogel to the subchondral bone with continuous drug release, thus achieving lesion positioning therapy. The screw‐threaded grooves held hydrogel well, and when the hydrogel inside the thread is crosslinked and properly air‐dried, its surface is below the contour of the ST‐needle, as shown above. While the hydrogel coating on the CA‐needle was blocked by the cartilage surface during the insertion, the hydrogel coating on the ST‐needle can reach the subchondral bone (**Figure** **4**Ai). After the ST‐needle reaches the subchondral bone, the loaded hydrogel absorbs moisture and expands in body fluids to firmly adhere to the surrounding subchondral bone. As the ST‐needle rotates and recedes, the hydrogel is left in the subchondral bone (Figure 4Aii).

To verify that our above mechanism was correct, We still used pig cartilage for in vitro experiment. First, the osteochondral cartilage was punctured using a CA‐needle loaded hydrogel (Figure 4Bi). As the photo showed, the hydrogel on the CA‐needle was blocked out by cartilage (the arrow pointed to hydrogel). Subsequently, we searched for hydrogel in subchondral bone by hard‐tissue slicing. histologic section showed that almost no hydrogel remained in the cavity (Figure 4Bii). We then used the ST‐needle loaded hydrogel to penetrate the pig cartilage. As shown in the photo, there was no obvious hydrogel blocked by cartilage outside the cartilage (Figure 4Biii). And a large amount of hydrogel was successfully retained in the subchondral bone in the histologic section (Figure 4Biv). Therefore, we demonstrated that the ST‐needle can effectively transport hydrogel to the subchondral bone.

Finally, we made an ST‐needle loaded with hydrogel with a fluorescence label (Cy‐7) and verified the effect of the ST‐needle transporting the hydrogel through the cartilage of isolated pig femurs (Figure 4C). As can be seen from the intensity of fluorescence, the fluorescence value of the tip of the CA‐needle after insertion into the cartilage and extraction of the needle was only reduced by 29.92% compared to the initial value, indicating that only a small amount of hydrogel was delivered to the cartilage. The fluorescence value of the ST‐needle reduced by 73.25% as compared with the initial value, indicating that the ST‐needle could better deliver the hydrogel to the subchondral bone (Figure 4E).

### 2.5 Cytotoxicity of Hydrogel and ST‐Needle

Because Lipo@DMA@HAMA was intended for clinical use, we tested its cytotoxicity. Lipo‐BAI and Lipo‐BAI@DMA@HAMA were co‐cultured with MSCs, and the MSCs were tested by performing a live/dead assay on days 1, 3, and 5 (Figure S6A, Supporting Information). The experimental results showed that the microscopic field of vision was full of living cells (stained green) and had very few dead cells (stained red) during the chosen three time periods. At the same time, the number of living cells increased significantly over time. We quantitatively analyzed the results of the live/dead assay by counting living cells (Figure S6B, Supporting Information). In addition, a CCK‐8 assay was performed using the same experimental design. The results of this assay showed that there was no significant difference in proliferation activity among the three groups at any time point (Figure S6C, Supporting Information). Taken together, these results suggested that these biomaterials had little cytotoxicity and may have good medical applications.

In the clinical practice of acupuncture treatment, the acupuncture needles are left in the body for ≈15–30 min. Therefore, the ST‐needle was also tested for its cytotoxicity. We took the tip of a sterile ST‐needle and co‐cultured it with MSCs for 1 h to simulate ST‐needles in vivo (Figure S7A, Supporting Information). Subsequently, we performed live/dead staining on the cells, and we observed that MSCs around the ST‐needle were all living cells stained green, and almost no dead cells stained red were seen. After the ST‐needle was removed, the MSC culture was continued for 24 h, 3, and 5 days, (Figure S7B–D, Supporting Information) and the live/dead cell staining was performed again. The cell density of the MSCs significantly increased compared with 24 h before, and almost no cells were stained red. Therefore, the ST‐needle has no toxicity to the surrounding cells and tissues are there are no toxic substances left after the ST‐needle is removed. In conclusion, our ST‐needle can be safely applied for clinical treatment.

### 2.6 Cellular and Molecular Mechanism of Hydrogel Transported by ST‐Needles in OA Treatment

**Figure 5:**
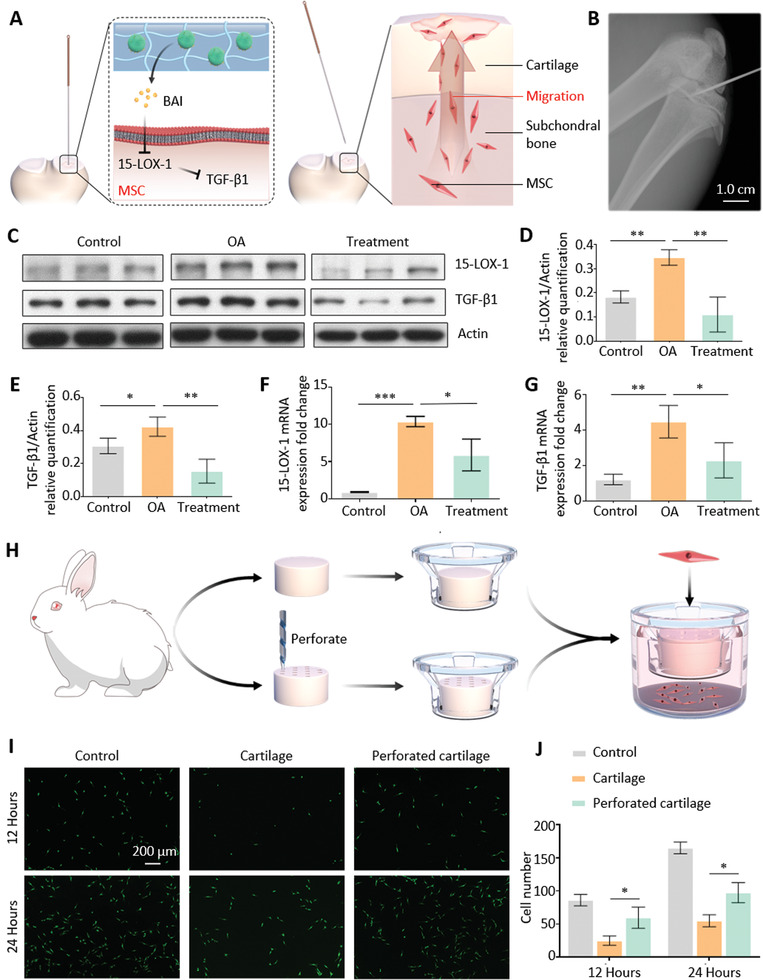
Cellular and molecular mechanisms of ST‐needle therapy for OA. A) Diagram of ST‐needles inhibiting cytokine synthesis and promoting stem cell migration. B) X‐rays showed that the ST‐needles accurately reached the subchondral bone. C) The Western blot of 15‐LOX‐1 and TGF‐*β*1. D–E) Quantitative analysis of the Western blot results of 15‐LOX‐1 and TGF‐*β*1. F,G) The PCR of mRNA of 15‐LOX‐1 and TGF‐*β*1. H) Schematic diagram of the experiment, in which the ST‐needle promoted MSC migration. I) Representative fluorescence images show live staining of the MSCs migrating through the cartilage. J) Quantitative analysis of live cells (*n* = 3). (* *P* < 0.05, ** *P* < 0.01, *** *P* < 0.001).

The mechanism of transporting hydrogel with ST‐needles for lesion positioning therapy in OA has two advantages: 1. After transporting the hydrogel to the subchondral bone with an ST‐needle, the hydrogel remains in the subchondral bone, continuously releases BAI, and regulates abnormal remodeling of the subchondral bone. 2. The ST‐needle leaves micron‐sized pores on the cartilage after extraction after treatment, which can serve as migration channels for MSCs, enabling MSCs of the subchondral bone to migrate to the surface of the cartilage and promote cartilage repair (**Figure** **5**A).

Previous studies have shown that one mechanism of abnormal remodeling of knee subchondral bone in OA patients is the abnormal secretion of 15‐the lipoxygenase‐1 (15‐LOX‐1) and TGF‐*β*1 cytokines.^[^ ^38^ ^]^ The overexpression of 15‐LOX‐1 in the weight‐bearing region of the joint can cause a synchronous increase in the expression level of TGF‐*β*1 in the subchondral bone, while an increase in the expression level of TGF‐*β*1 can lead to an increase in abnormal cell phenotypes and excessive differentiation of MSCs, leading to abnormal bone remodeling and increased sclerosis of the subchondral bone. Therefore, we investigated whether lesion positioning therapy with ST‐needles can inhibit the secretion of cytokines in subchondral bone abnormalities. We established an OA rat model and set up a Control group, a model group (OA group), and a treatment group treated with ST‐needles (Figure 5B). Subsequently, the cells of the rat subchondral bone were extracted, and protein and mRNA were extracted for detection by Western blot and Polymerase chain reaction (PCR), respectively (Figure 5C–G). We found that the mRNA and protein levels of 15‐LOX‐1 and TGF‐*β*1 were significantly up‐regulated in the subchondral bone of rats in the OA group compared with the Control group. This shows that OA leads to abnormal secretion of subchondral bone cytokines. After treatment with ST‐needles loaded with Lipo‐BAI@DMA@HAMA, the mRNA and protein levels of 15‐LOX‐1 and TGF‐*β*1 were significantly lower than those of the OA group. Thus, precision therapy with ST‐needles can effectively reverse the secretion of abnormal cytokines.

To verify that the micropores left after ST‐needle treatment enhance MSC migration, we designed a cell migration experiment (Figure 5H). An ST‐needle was used to puncture dozens of perforated pores in the cartilage disc of the knee joint of rabbits to simulate cartilage treated with ST‐needles, used as the perforated cartilage group. Meanwhile, intact cartilage discs that had not been punctured by ST‐needles were used as the cartilage group. Subsequently, we placed the two types of cartilage separately into Transwell assays and added MSCs and culture media according to the Transwell instructions. We set two time points, 12 and 24 h, to stain the cells (Figure 5I). The cells in the cartilage group had fewer migratory cells than those in the Control group, which were blocked by cartilage discs. Due to the micropores left in the cartilage disc by the ST‐needle, the number of cells in the perforated cartilage group that were transported was significantly higher than that of the cartilage group (Figure 5J). In conclusion, the micropores produced by ST‐needle treatment can effectively facilitate the migration of MSCs from the subchondral bone to the chondral surface.

### 2.7 Lesion Positioning Therapy with ST‐Needles Can Alleviate Subchondral Bone Lesions

**Figure 6:**
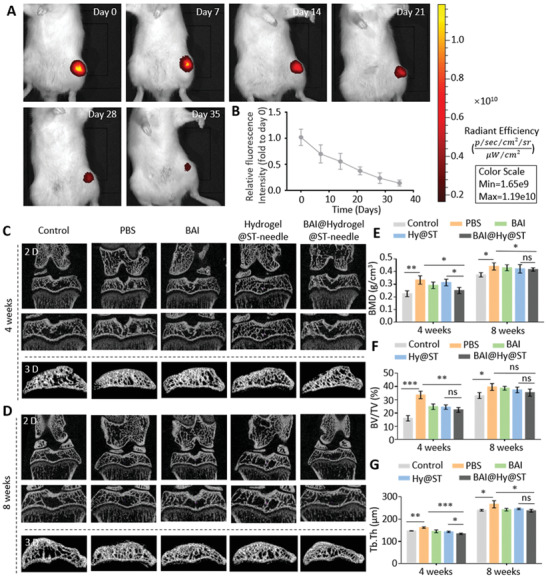
Using ST‐needles to treat OA rats. A) Cy‐7‐labeled hydrogel was used to verify that the hydrogel could be left in rats for a long time to release the drug continuously. B) Quantitative analysis of fluorescence intensity *(n* = 3). Micro‐CT showed the subchondral bone of rats at C) 4 weeks and D) 8 weeks. E–G) Quantitative analysis of the BMD, BV/TV, and Tb.Th of the subchondral bone (*n* = 3). (ns: no significance, * *P* < 0.05, ** *P* < 0.01, *** *P* < 0.001).

To study the effect of Lipo@DMA@HAMA on long‐term retention in subchondral bone, we labeled Lipos with fluorescent dye (Cy‐7). Labeled Lipo@DMA@HAMA was transported to the subchondral bone via the ST‐needle, and fluorescence imaging was performed with the IVIS‐spectrum system (Xenogen, Hopkinton, MA, USA) for 5 consecutive weeks (**Figure** **6**A). We also used the simple fluorescent dye (Cy‐7) for joint cavity injection as a control. The fluorescence intensity of the Control group decreased sharply in the first 2 days, and the fluorescence intensity measured on the second day was only about one hundredth of that on the first day, indicating that the retention effect of the free drugs in the joint cavity was weak (Figure S8, Supporting Information). On the contrary, the fluorescence intensity of the Lipo@DMA@HAMA hydrogel decreased very slowly, only ≈50% by the third week, and the fluorescence signal remained for more than 1 month, indicating that the Lipo@DMA@HAMA hydrogel had an excellent retention effect in subchondral bone, ensuring the long‐term sustained release of BAI.

To further verify the efficacy of the lesion positioning therapy of the ST‐needle on OA, an OA rat model was established by joint cavity injection of iodoacetic acid,^[^ ^39^, ^40^ ^]^ and a blank control was used as the Control group. After successful modeling, we injected PBS (PBS group) and BAI (BAI group) into the articular cavity and used ST‐needles loaded with drug‐free hydrogel for lesion positioning therapy in OA rats (Hydrogel@ST‐needle group). Also, use ST‐needle loaded with drug‐containing hydrogel for lesion positioning therapy in OA rats (BAI@Hydrogel@ST‐needle group). Based on the comparison of BAI@Hydrogel@ST‐needle group with BAI group and Hydrogel@ST‐needle group, the using of ST‐needle system to deliver drugs can be rigorously verified by controlling variables to significantly improve efficacy. Micro‐CT is currently widely used to measure bone tissue. Compared with the limitations of 2D images, micro‐CT can perform X‐ray scanning and 3D reconstruction of bone tissue, which can allow more intuitive observation and measurement of bone density, bone morphology, bone trabecular structure, and other data.^[^ ^41^ ^]^ In this study, we sampled at 4 and 8 weeks, and 3D models of the knee joint of rats were obtained by micro‐CT scanning of the subchondral bone (Figure 6C,D). We also measured 3D‐related parameters of the subchondral bone (Figure 6E–G). Compared with the Control group, bone mineral density (BMD), bone fraction volume (BV/TV), and bone trabecular thickness (Tb.Th) in PBS‐treated group increased statistically significantly. BMD, BV/TV, and Tb.Th of the BAI‐treated group were lower than those of the PBS group. Meanwhile, BMD, BV/TV, and Tb.Th of the ST‐needle group were significantly lower than those of the PBS group. Meanwhile, we compared the data of “Hydrogel@ST‐needle group” and “BAI@Hydrogel@ST‐needle group,” and found that whether at 4 or 8 weeks, the measured value of each sample of " BAI@Hydrogel@ST‐needle group" was less than the minimum value of " Hydrogel@ST‐needle group". In addition, BMD and Tb.Th values were statistically different. This suggests that the efficacy of “BAI@Hydrogel@ST‐needle group” was superior to “Hydrogel@ST‐needle group.” However, the fluctuation range of data related to physiological structure was relatively small, and the basic value of each index increased greatly due to the development of bone after rats were fed to 8 weeks. Therefore, there was no statistically significant difference at week 8. In conclusion, lesion positioning therapy of subchondral bone in OA rats with ST‐needles significantly improves the abnormal remodeling of the subchondral bone.

### 2.8 Lesion Positioning Therapy with ST‐Needles Improves Cartilage Lesions

**Figure 7:**
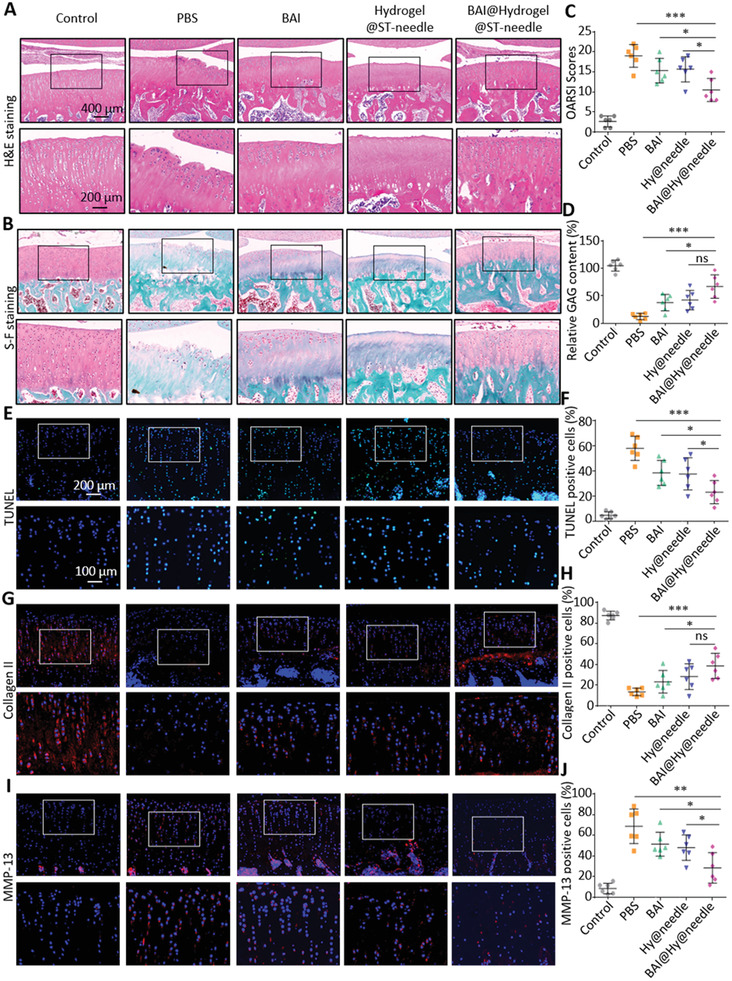
Treatment reduced cartilage degeneration in OA rats (*n* = 6 in each group). A) Representative images of H&E staining and B) OARSI scores of articular cartilage in each group. C) Representative images of Safranin O‐fast green staining showing the histological changes of the cartilage in five groups. D) Relative glycosaminoglycan content in each group. E) Representative sections of TUNEL staining for apoptotic cells. F) Quantification of TUNEL‐positive cells. G) Representative sections of immunofluorescence staining of collagen II. H) Quantification of collagen II‐positive cells. I) Representative sections of immunofluorescence staining of MMP‐13. J) Quantification of MMP‐13‐positive cells. (ns: no significance, * *P* < 0.05, ** *P* < 0.01, *** *P* < 0.001).

We further verified that lesion positioning therapy with ST‐needles significantly improves the pathological changes of cartilage after improving abnormal remodeling of OA subchondral bone. We established healthy rats as “Control group” and we injected PBS (PBS group) and BAI (BAI group) into the articular cavity and used ST‐needles loaded with drug‐free hydrogel for lesion positioning therapy in OA rats (Hydrogel@ST‐needle group). Also, use ST‐needle loaded with drug‐containing hydrogel for lesion positioning therapy in OA rats (BAI@Hydrogel@ST‐needle group). Based on the comparison of BAI@Hydrogel@ST‐needle group with BAI group and Hydrogel@ST‐needle group, the using of ST‐needle system to deliver drugs can be rigorously verified by controlling variables to significantly improve efficacy. We carried out animal experiments on the knee joints of rats during the eighth week of the experiment and prepared paraffin‐embedded sections for morphological staining and immunofluorescence detection. Hematoxylin and eosin (H&E) staining (**Figure** **7**A) and Safranin O‐fast green staining (Figure 7B) showed the presence of significant OA features (surface irregularities and erosion cracks). These characteristics were most obvious in the PBS group, indicating that OA modeling was very successful. Whereas, in the BAI group, these characteristics were not as obvious as those in the PBS group, and the cartilage morphology of the BAI@Hydrogel@ST‐needle was the best. The morphological changes of the cartilage were quantitatively analyzed using the OARSI score (Figure 7C). The OARSI score of the PBS group decreased the most, whereas the OARSI score of the treatment group was better than that of the PBS group, among which the BAI@Hydrogel@ST‐needle group had the best effect, which decreased by 44.74%, followed by the BAI group, which decreased by 19.32%. Moreover, the OARSI score of BAI@Hydrogel@ST‐needle group was significantly lower than that of Hydrogel@ST‐needle group, further verifying that ST‐needle system could bring better efficacy. The results of the Safranin O‐Fast Green staining were also quantitatively analyzed, and the content of glycosaminoglycan (red staining) represented the health degree of the cartilage matrix (Figure 7D). Among them, the content of glycosaminoglycan was the highest in the BAI@Hydrogel@ST‐needle group (66.90%), followed by the BAI group (37.77%), indicating that lesion positioning therapy of ST‐needle had a good effect on relieving cartilage lesions. There was a large difference between the average values of BAI@Hydrogel@ST‐needle group and Hydrogel@ST‐needle group, but we were surprised to find that there was no statistical difference. The reason may be that the accumulation of glycosaminoglycan took time. Due to the limited duration of our experiment, there was no statistical difference between the two groups, although there was a large gap between the average values.

Subsequently, TUNEL staining was used to detect chondrocyte apoptosis (Figure 7E). The BAI@Hydrogel@ST‐needle group had the least apoptotic cells (green staining), followed by the BAI group. Figure 7F shows the quantification of positive stained cells. Compared with the PBS group, the apoptosis rate of the BAI@Hydrogel@ST‐needle group was the lowest and decreased by 61.31%, whereas that of the BAI group decreased by 40.65%. Meanwhile, the apoptosis rate of chondrocytes in BAI@Hydrogel@ST‐needle group was significantly lower than that in Hydrogel@ST‐needle group, confirming that ST‐needle system could significantly reduce the damage of chondrocytes caused by OA. In addition, immunofluorescent staining was used to detect the expression of collagen II and MMP‐13, the main biomarkers of cartilage (Figure 7G–J). For determining the expression level of collagen II in all treatment groups, the Control group was used as the baseline and it was noted that the expression level of collagen II (stained red) in all treatment groups decreased, among which the BAI@Hydrogel@ST‐needle group had the smallest decrease, followed by the BAI group. The number of positively stained cells is shown in Figure 7H. By using the PBS group as the baseline, it was determined that the collagen II expression level in the BAI@Hydrogel@ST‐needle group was the highest, with an increase of 186.27%, followed by the BAI group, with an increase of 72.38%. MMP‐13 was the main enzyme targeting cartilage degradation, which can degrade not only collagen II in cartilage, but also proteoglycans, type IV and IX collagen, osteonectin, and basement membrane proteoglycans in cartilage.^[^ ^42^ ^]^ Therefore, MMP‐13 plays an important role in OA. The expression level of MMP‐13 in the PBS group increased significantly, which also led to the degradation of the cartilage matrix in OA and the progression of the disease. By using the PBS group as the baseline, it was determined that the expression level of MMP‐13 in the BAI@Hydrogel@ST‐needle group decreased by 58.42%, followed by the BAI group, which decreased by 25.14% (Figure 7J). What's more, we also compared BAI@Hydrogel@ST‐needle group and Hydrogel@ST‐needle group, and the average expression level of collagen II in BAI@Hydrogel@ST‐needle group was much higher than that in Hydrogel@ST‐needle group, while the expression level of MMP‐13 was much lower than that in Hydrogel@ST‐needle group. We found no statistical difference between the data of collagen II, the reason may be the same as mentioned in the analysis of glycosaminoglycan. The MMP‐13 data were statistically different. Therefore, we verified that the ST‐needle system can significantly improve the role of chondrocytes in repairing cartilage, so as to better treat OA.

In conclusion, these results suggest that lesion positioning therapy with ST‐needles can significantly inhibit the abnormal remodeling of subchondral bone, thereby alleviating cartilage injury and degeneration, compared with treatment with BAI injection. This provides an attractive strategy for the treatment of OA.

## 3 Conclusion

In this study, we innovatively designed a CA‐needle with a screw‐thread structure at the tip, which can robustly break through physical barriers and achieve minimally invasive and lesion positioning in lesions. The screw‐thread loaded hydrogel was realized through a polymer interface interaction, and the thread groove structure and dry‐wet transformation of the hydrogel were used to successfully transport the drug‐loaded gel into the lesion and continuously release the drug. Briefly, the dopamine‐modified hydrogel firmly adhered to the thread groove of the ST‐needle, and under the protection of the thread groove was precisely delivered to the lesion requiring treatment. The hydrogel absorbed body fluids, swelled, squeezed, and adhered to the surrounding tissues, and thus remained within the lesion, continuously releasing the drug to achieve lesion positioning therapy. The significant efficacy of lesion positioning therapy with the ST‐needle was further verified in an OA rat model, in which precise positioning was achieved in the subchondral bone, inhibiting the secretion of abnormal cytokines, promoting the migration of MSCs to cartilage defects, and repairing the cartilage. Overall, our ST‐needle and hydrogel system can greatly expand the clinical application of precision positioning therapy.

## 4 Experimental Section

### Materials

Conventional experimental reagents and consumables were from Sigma Aldrich. The special reagents used in the experiment are shown below. HA, N‐[2‐(3,4‐Dihydroxyphenyl)ethyl]‐2‐methylacrylamide, and BAI were obtained from Macklin Co., Ltd. (Shanghai, China). Cholesterol, lecithin (from egg yolk), and MIA were obtained from Sangon Biotech (Shanghai) Co., Ltd. (Shanghai, China). CA‐needles were obtained from Yuwell Co., Ltd. (Shanghai, China).

### Preparation of ST‐Needle

A professional metal processing factory was found to produce ST‐needles (Figure S9, Supporting Information). After repeated experiments, 314 medical stainless steels were finally chosen as the material of ST‐needle. The diameter of the ST‐needle was 0.25 mm, the pitch was 0.9 mm, and the groove depth was 0.05 mm. Of course, these parameters can be changed according to actual requirements.

### Preparation of Lipos

The Lipos were prepared according to the previously reported thin‐film dispersion method.^[^ ^43^ ^]^ In a nutshell, 60 mg of lecithin and 20 mg of cholesterol were dissolved in 10 mL of chloroform. The mixture was added to a round‐bottomed flask and the organic solvent was allowed to evaporate in a rotary evaporator to form a lipid film attached to the bottom of the flask. Then, 3 mL of distilled water was added to the flask and the lipid membrane was dissolved in water by gently shaking. Finally, a strong probe ultrasonic solution (60 mono‐pulse min^−1^, 130 W) was used. After 5 min, evenly dispersed Lipos were obtained. In order to prepare fluorescent liposomes, DSPE‐PEG‐Cy7 (Catalog No. R‐DPC7‐2000) was purchased from Ruixi Biotechnology Co., Ltd. (Xian, China), and added to the organic phase at a concentration of 5% (w/v). The subsequent preparation steps were the same as above. Finally, the Cy7‐labeled liposomes were prepared and subsequently used to synthesize fluorescently labeled Lipo@DMA@HAMA.

### Synthesis of HAMA and Lipo@DMA@HAMA

The synthesis steps of HAMA were the same as those mentioned in the previous papers.^[^ ^44^ ^]^ In brief, PBS was preheated to 60 °C and then HA was added to control the concentration at 10% (w/v). After HA was completely dissolved, methacrylic acid (MA) solution was added and stirred continuously for 1 h to allow complete reaction of HA and MA. Then, dialysis was performed for a week to remove the impurities (in a 14‐kDa cut‐off analysis tube). Finally, HAMA was obtained, which can be preserved for a long time after freeze‐drying.

An appropriate amount of HAMA freeze‐dried powder and DMA powder dissolved in distilled water was used to control the HAMA concentration at 3–5% and the DMA concentration at ≈1%. These were fully dissolved and stirred evenly, and 1% Lipos were added to the mixed solution to prepare Lipo@DMA@HAMA. The hydrogel was cured by UV crosslinking at a 350 nm wavelength to form a solid hydrogel loaded with Lipos with adhesive function.

### Lipo@DMA@HAMA Adhesion ST‐Needle Test

The prepared DMA@HAMA solutions of ≈0.2 mL were taken and placed them at the bottom of centrifugal tubes. The tip of the ST‐needle was inserted between the hydrogel and UV light was irradiated to solidify the hydrogel. Then, the DMA@HAMA hydrogel with adhesion stuck to the ST‐needle, and when the ST‐needle was lifted, it was not separated from the hydrogel but lifted up the hydrogel and the centrifugal tube together.

### Degradation and Swelling Test

The hydrogel was immersed in PBS containing collagenase to evaluate the degradability. In brief, the hydrogel (30 mg) was suspended in the collagenase solution (1 U mL^−1^ in PBS, 1 mL, pH = 7.4) and placed in a shaking incubator (80 rpm, 37 °C). Every 2 days, the supernatant was replaced with fresh hyaluronidase solution. At the specified time point, the residual weight of MGs was measured and was compared to the initial weight. In addition, the morphological changes of hydrogel were monitored by microscopic observation. For the swelling test, 3 mg of hydrogel were added into the 1.5 mL tube, and the weight of the MGs and the tube was measured before 1 mL deionized water was added. The pH of the suspension was adjusted to 7.4, and the tube was placed in a shaking incubator at 37 °C and 80 rpm. At defined time points, the tubes were centrifuged (3000 rpm, 3 min) before removing the supernatant. Excess water was blotted using filter paper before weighting the hydrogel. The whole procedure was repeated until a constant weight was obtained.

### Morphological Characterization

The shapes of the CA‐needle and ST‐needle were filmed with a SONY macro lens (Camera: SONY A7*α*2, Macro lens: SONY 90f 2.8). The size of the Lipos was accurately measured by dynamic light scattering (Zetasizer Nano S, Malvern, UK). Finally, the shape of the Lipos was observed using a transmission electron microscope (Tecnai G2 Spirit Biotwin, US).

### In Vitro Release Profile

The BAI concentrations of Lipo‐BAI and Lipo‐BAI@DMA@HAMA were adjusted to 200 µm. Then, 1 mL suspension in PBS solution containing Lipo‐BAI or Lipo‐BAI@DMA@HAMA was loaded onto the filter membrane (14 kDa) after sealing the bag mouth. This bag was loaded into a 50 mL centrifuge tube and 20 mL of PBS was added. The centrifuge tube was placed on a shaker and the sample was shaken at 37 °C. At the set time point, 1 mL of extracapsular fluid was collected and the same amount of PBS was added. The composition of BAI in the aqueous solution was measured using a UV/Visible Photometer‐5100 (METASH, China).

### MSC Isolation and Culture

MSCs (cat. no. RASMX‐01001) were purchased from Cyagen Biosciences (Guangzhou, China). When the cells reached ≈70% confluency in the flask, trypsin was used to separate the cells from the flask and performed subculture. After culturing cells for 3–5 generations, they were used for subsequent experiments.

### Cell Cytotoxicity

The CCK‐8 assay was used to detect the cytotoxicity of Lipo‐BAI@DMA@HAMA and Lipo‐BAI on MSCs. The right number of cells was planted in 96‐well plates according to the growth rate of the MSCs and different biomaterials were added to different groups according to the experimental design. These plates were then incubated in a cell incubator for different periods of time. Then, according to the instructions of the CCK‐8 kit (Beyotime, China), the reagent was prepared and added to the cells. Finally, the cells were again placed into the cell incubator and incubated for 2 h. The OD value of the solution was measured at 450 nm using a microplate reader. The live/dead cell kit (Invitrogen, UK) was used to look at the number of living and dead cells. The procedure was basically the same as the above experiment, except that the cells in this experiment were grown in 48‐well plates, which were easy to observe under a microscope. After adding reagents of the live/dead cell kit, the cells were incubated in a cell incubator for half an hour. Finally, the cells were examined under a fluorescence microscope (PCOM, Nikon, Japan). Living cells stained green and dead cells stained red. The cytotoxicity test of the ST‐needles was basically the same as the appellate test, and the MSCs of suitable density were also grown in 48‐well plates. The tip of the ST‐needle was disinfected, and 48‐well plates were filled with co‐culture with cells. After 1 h of co‐culture, some cells were directly subjected to the live/dead cell staining experiment as described above, and the other cells were cultured for 24 h after ST‐needle removal, and then subjected to the live/dead cell staining experiment.

### CA‐Needle and ST‐Needle Penetration Test of Cartilage

The femoral condyle, tibial plateau, and talus were dissected from the hind limbs of domestic pigs. CA‐needles and ST‐needles were used to conduct puncture experiments on the cartilage of the three sites using acupuncture and moxibustion to simulate clinical practice. Meanwhile, a stopwatch was used to record the puncture time. Then, using a small hand‐held cutting machine, the cartilage was cut to expose the acupuncture needles inside, and the thickness of the cartilage and the depth of the acupuncture needle penetration into the cartilage were measured.

### CA‐Needle and ST‐Needle Transport Hydrogel Test

The femoral condyles of pigs were used for this experiment. A CA‐needle and an ST‐needle were modified with Lipo@DMA@HAMA labeled with fluorescent dye (Cy‐7). An IVIS‐spectrum system (Xenogen, Hopkinton, MA, USA) was used to detect and record the initial fluorescence intensity on the tip of the two microneedles. Subsequently, the CA‐needle and the ST‐needle were used to puncture the cartilage, and the hydrogel was transported into the subchondral bone by the two microneedles. Finally, the fluorescence intensity of the cartilage surface and the tips of the two kinds of microneedles were measured. Considering the depth of the cartilage sample, the cartilage surface was much deeper than the position of the microneedle, and the height of the cartilage surface itself fluctuated greatly, which could produce a large error. Therefore, the fluorescence intensity values of the cartilage surface and the microneedle tip were not added or subtracted directly. The initial fluorescence intensity value of the microneedle tip minus the residual fluorescence intensity value of the microneedle tip after the experiment was used to obtain the fluorescence intensity of the cartilage surface.

### Western Blot Test

A radio immunoprecipitation assay (RIPA lysis buffer) was used to extract total protein from tissues. A bicinchoninic acid kit (Thermo Scientific, USA) was used to detect the concentration of the extracted proteins. The glass plate, plastic plate, and electrophoresis tank of the SDS‐PAGE glue were used to form an electrophoresis device. After pouring the electrophoresis solution, the comb was slowly pulled out. According to the protein sample type and the measured protein concentration, the protein sample loading quantity was determined. The power supply of the electrophoresis tank was adjusted to 55 V at constant pressure and the voltage to was adjusted to 110 V at constant pressure according to the downward diffusion of the marker and bromophenol blue (generally, the marker was initially diffused, ≈30 min) until the marker corresponding to the desired target protein was fully diffused and available for glue cutting. A primary antibody diluent was configured according to the relevant antibody instructions (ab244205, ab215715, ab8226, Abcam, UK), completely immersed the PVDF membrane in the liquid, and incubated overnight in a refrigerator at 4 °C. The SDS‐PAGE glue glass plate, plastic plate, and electrophoresis tank were used to form the electrophoresis device, and the electrophoresis solution was poured in, and the comb was slowly pulled out. The second antibody was mixed with TBST solution containing 5% skim milk in a ratio of 1:15 000. The prepared liquid was poured into the dish, the PVDF membrane was put into the dish, and then the dish was shaken at room temperature for 2 h (the shaker speed was 50 times min^−1^). Then choose the appropriate exposure mode and exposure time for exposure.

### PCR Test

At 4 °C, Trizol was used to digest and lyse the total RNA from the tissues. The lysate was pipetted into a 1.5 mL centrifuge tube, 0.2 mL of chloroform was added to each tube, and then it was shaken for 15 s and incubated on ice for 15 min, followed by centrifugation (4 °C, 15 min, 12 000 × *g*). After centrifugation, the centrifuge tube was divided into three layers: a transparent and colorless RNA‐containing upper layer, DNA in the white layer, and protein in the red layer. The colorless and transparent RNA in the upper layer was carefully absorbed and transferred into a new centrifuge tube with a pipette. An equal volume of isopropyl alcohol was added to the new centrifuge tube, and after mixing, it was placed on ice for another 15 min, then centrifuged (4 °C, 1200 × *g*, 10 min). The supernatant was removed and was washed with 75% ethanol. Finally, the NanoDrop system was used to detect the concentration of the extracted RNA. RNA was reversed‐transcribed into cDNA, and PCR amplification was performed according to the instructions of the reverse transcription kit (No. RR82LR, Takara, USA) and the RT‐PCR kit (No. RR036A, Takara, USA). Specific genes and primer sequences are shown in Table S1, Supporting Information.

### Cell Migration Test

The Transwell system (8 µm tin, Corning, USA) was used to demonstrate the migration of MSCs. In brief, MSCs (5 × 10^4^) were added after the upper chambers were treated with different cartilage discs. Basal medium containing serum was added to the lower chambers. Two time points, 12 and 24 h were set up, for fluorescent staining of living MSCs in the lower chambers. Finally, the cells were observed and counted with a fluorescence microscope.

### Construction of the OA Model

The animal experiment was approved by the Animal Research Committee of Shanghai Jiaotong University School of Medicine (SYXK 2018‐0027). The OA model of rats was established by injecting iodoacetic acid into the joint cavity. In brief, SD rats were commercially purchased and were raised for a week in an animal laboratory to allow them to adapt to the new environment. Subsequently, the rats were anesthetized with chloral hydrate (400 mg kg^−1^) and then injected with iodoacetic acid (2.5 mg) into the joint cavity after successful anesthesia. 3 days after the injection, follow‐up experiments were conducted.

### Detection of the Retention Ability of Hydrogel in Lesions

Lipo@DMA@HAMA was labeled with fluorescein by carrying Cy‐7 in Lipos. After the hydrogel was delivered to the subchondral bone using the ST‐needle, fluorescence was detected using the IVIS‐spectrum system (Xenogen, Hopkinton, MA, USA) at an excitation wavelength of 750 nm and an emission wavelength of 773 nm. The rats were tested at several time points in the experiment design and the fluorescence intensity data were obtained, which were converted according to the initial fluorescence intensity.

### X‐Ray and Micro‐CT Test

After receiving acupuncture treatment, the rats received X‐ray radiography (Faxitron X‐ray, USA) and high‐resolution micro‐CT (SkyScan 1172, Belgium). The data collection time was set as 4 and 8 weeks after the beginning of the experiment and conducted quantitative analysis on the collected images. The osteophyte volume was calculated according to the 3D‐reconstructed micro‐CT results.^[^ ^45^ ^]^

### Histological and Immunofluorescence Evaluation

After completing the animal experiment according to the experimental design, the rats were killed to obtain the samples of knee joint. After conventional decalcification and paraffin embedding, the samples were cut into 5‐micron thick slices using a slicer. In order to observe the morphology of tissue sections, H&E staining and Saffron O‐fast Green staining were performed routinely. For quantitative analysis of the morphology, the OARSI score system was used.^[^ ^46^ ^]^ Three experimenters were selected to score each section independently according to the scoring standard of OARSI scores. The paraffin sections were processed and then put into EDTA antigen repair solution in a microwave oven at medium heat for 5 min, rested for 5 min, and low heat for 10 min. During the repair process, the slices were not dried to prevent excessive evaporation and loss of buffer solution. After natural cooling, the slides were put into PBS and shaken three times in a shaker, each time for 5 min. The slides were placed in 3% H_2_O_2_ and incubated at room temperature, away from light, for 25 min. The slides were placed in PBS and shaken three times in a shaker, each time for 5 min. Then, 3% sealing solution was added, the tissue samples were covered evenly, and sealed at room temperature for 30 min. The sealing fluid in the previous step was removed, and the primary antibodies of collagen II and MMP‐13 (ServiceBio, China) diluted with PBS were dropped onto the sections, and the TUNEL kit (ServiceBio, China) was added according to the instructions. The samples were incubated at 4 °C overnight. The incubated primary antibody specimens were put into PBS and placed on the shaker three times for 5 min each time. After drying the specimens, the corresponding secondary antibody was added to cover the specimens, and they were incubated for 90 min at room temperature. The incubated second antibody specimens were placed in PBS on the shaker and were shaken three times for 5 min each time. After the samples were dried, the chromogenic agent was added, and staining was observed under the microscope. When the staining was completed, the staining was stopped by washing with ultra‐clean water. The processed specimens were placed in 75% alcohol for 5 min, 85% alcohol for 5 min, anhydrous ethanol for 5 min, and xylene for 5 min. The specimens were then removed from xylene to dry, and were sealed with neutral gum.

### Statistical Analysis

All data are indicated by mean ± SD, and the difference between groups was analyzed by one‐way ANOVA with Bonferroni test for multiple comparisons. At least three independent samples were contained in each group for each statistical analysis. Column statistics were performed on datasets to check for normal distribution. Statistical analyses were performed using Excel (Microsoft, USA), SPSS (IBM Corp, USA), and GraphPad Prism 9 (GraphPad Software, USA). *P* < 0.05 was considered to be statistically significant.

## Conflict of Interest

The authors declare no conflict of interest.

## Supporting information

Supporting Information

## Data Availability

Research data are not shared.
