# Correction to “Transporting Hydrogel via Chinese Acupuncture Needles for Lesion Positioning Therapy”

**DOI:** 10.1002/advs.202309934

**Published:** 2024-03-13

**Authors:** Feng Lin, Zhen Wang, Lei Xiang, Longxi Wu, Yupu Liu, Xiaobing Xi, Lianfu Deng, Wenguo Cui

Transporting Hydrogel via Chinese Acupuncture Needles for Lesion Positioning Therapy, *Advanced Science* **2022**, *9*(17), https://doi.org/10.1002/advs.202200079.

In the originally published paper, in Figure S7 (Supporting Information), due to the computer lag caused by the large amount of processing fluorescence data for live/dead staining of cells, we accidentally copied the wrong pictures for the final image layout by mistake. As a result, Figure S7C,D are the wrong pictures. And we have replaced them with the correct ones, the new Figure S7 is shown below:

**Figure S7:**
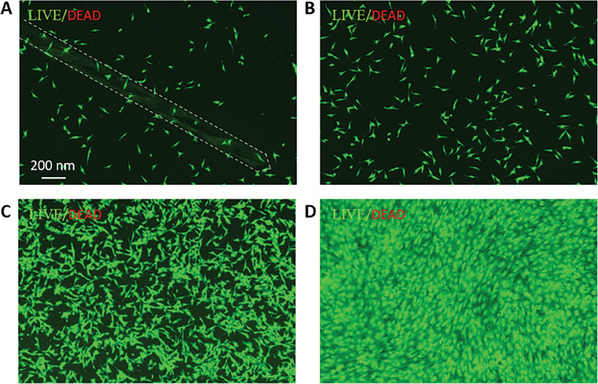
Cytotoxicity of ST‐needle. A) The results of live/dead staining about MSC co‐cultured with ST‐needle for 1 h (The white dotted line shows the outline of the ST‐needle). After the ST‐needle were removed from the culture medium, the MSC were cultured for 24 h (B), 3 days (C), 5 days (D), and then tested with live/dead staining.

We apologize for this error.

