# Supplementary material for: Transporting Hydrogel via Chinese Acupuncture Needles for Lesion Positioning Therapy
Source: Adv Sci (Weinh). 2022 Apr 11;9(17):2200079. doi: 10.1002/advs.202200079 (PMC9189641; doi:10.1002/advs.202200079)
Supplement: Supplementary file 1 — Supporting Information [file ADVS-9-2200079-s001.pdf]

ADVANCED SCIENCE
Open Access

Supporting Information

for *Adv. Sci.*, DOI 10.1002/advs.202200079

Transporting Hydrogel via Chinese Acupuncture Needles for Lesion Positioning Therapy

*Feng Lin, Zhen Wang, Lei Xiang, Longxi Wu, Yupu Liu, Xiaobing Xi, Lianfu Deng and Wenguo Cui**

Supporting Information

Transporting Hydrogel *via* Chinese Acupuncture Needles for Lesion Positioning Therapy

*Feng Lin, Zhen Wang, Lei Xiang, Longxi Wu, Yupu Liu, Xiaobing Xi, Lianfu Deng, Wenguo Cui**

Dr. F. Lin, Dr. Z. Wang, Dr. L. Xiang, Dr. L. Wu, Dr. Y. Liu, Prof. X. Xi, Prof. L. Deng, Prof. W. Cui
Department of Orthopaedics, Shanghai Key Laboratory for Prevention and Treatment of Bone and Joint Diseases, Shanghai Institute of Traumatology and Orthopaedics, Ruijin Hospital, Shanghai Jiao Tong University School of Medicine, 197 Ruijin 2nd Road, Shanghai 200025, P. R. China.

E-mail address: wgcui80@hotmail.com; wgcui@sjtu.edu.cn (W. Cui).

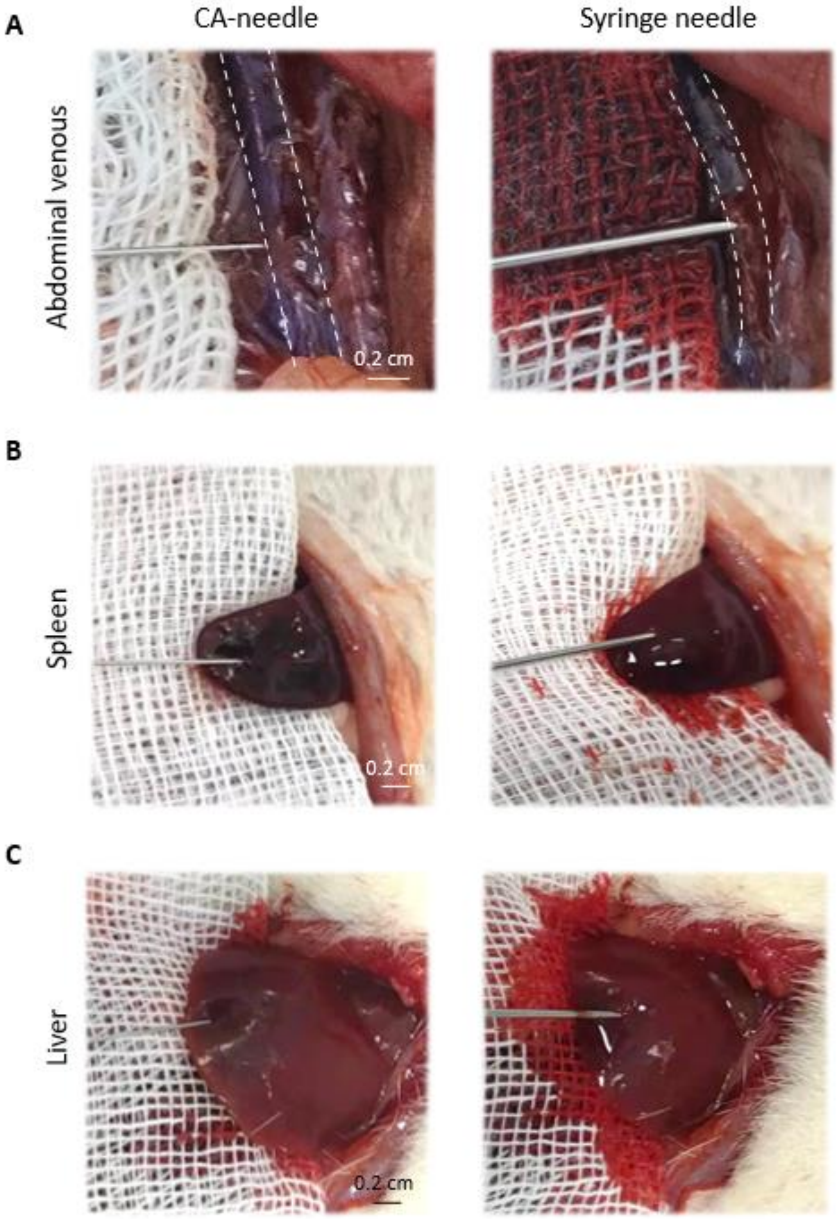

Figure S1. Compared to syringe needle, Chinese acupuncture needle causes less damage to tissues and organs. A) Abdominal venous. B) Spleen. C) Liver.

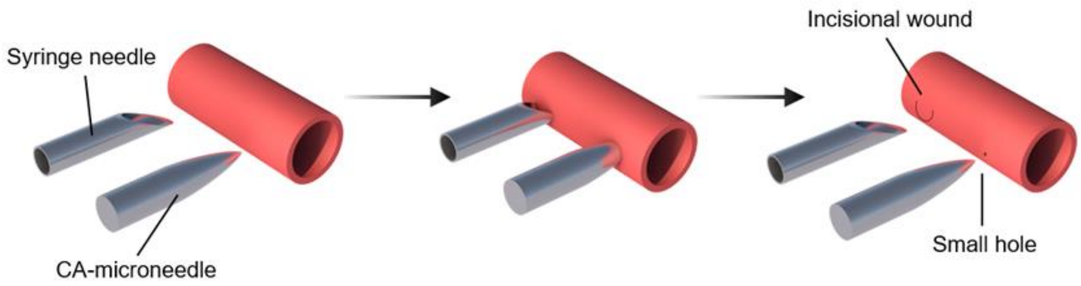

Figure S2. Schematic diagram of the mechanism by which CA-needles are minimally invasive.

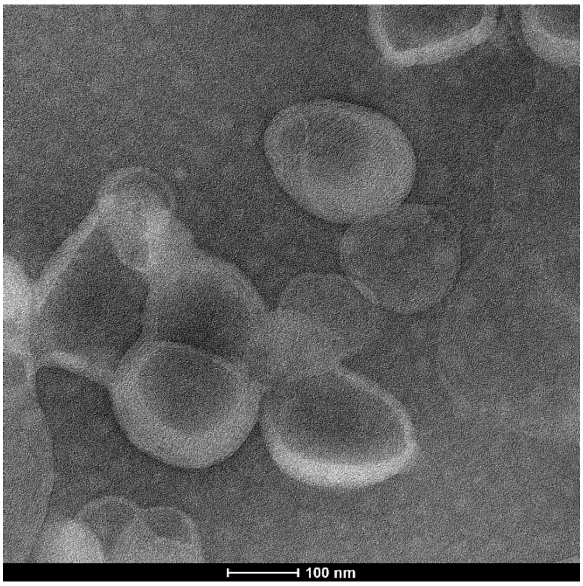

Figure S3. The TEM micrograph of multiple nanoparticles.

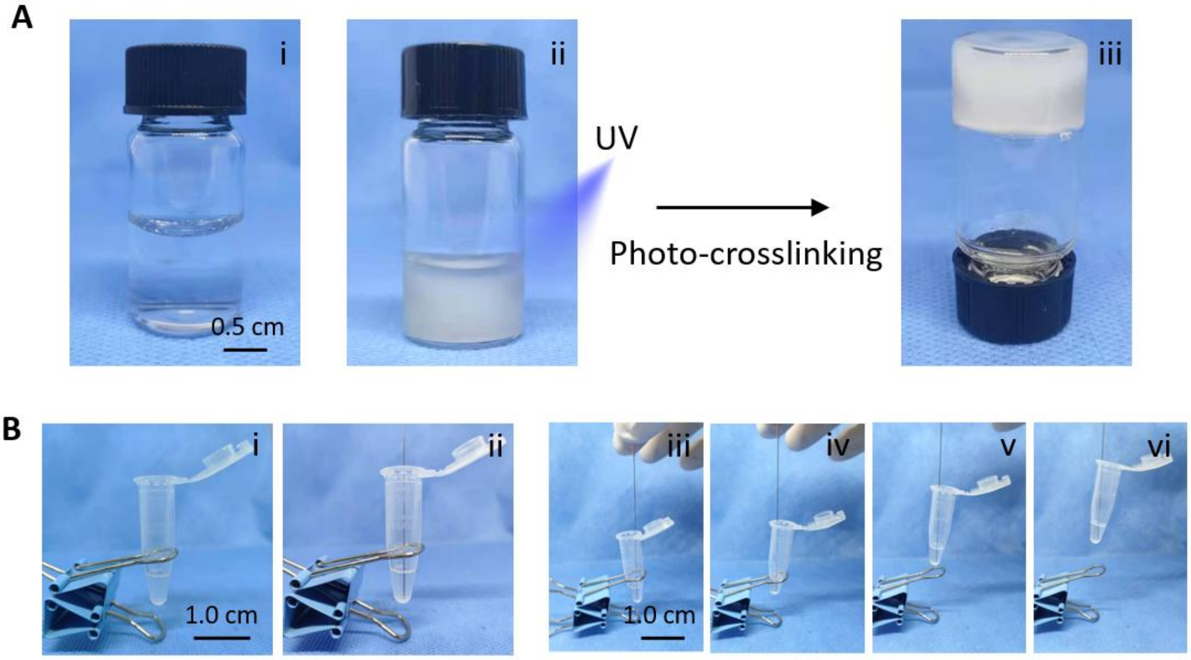

Figure S4. Characteristics of Lipo@DMA@HAMA hydrogel. A) Photograph of the aqueous solution of HAMA (i), aqueous solution of Lipo@DMA@HAMA (ii), Photo-crosslinked Lipo@DMA@HAMA hydrogel (iii). B) Hydrogel adheres to the metal surface. (i) Placed the DMA@HAMA in the centrifuge tube. (ii) The ST-needles were inserted into the hydrogel and photo-crosslinked. (iii-vi) Lifting the ST-needle, DMA@HAMA was found to adhere to the ST-needle.

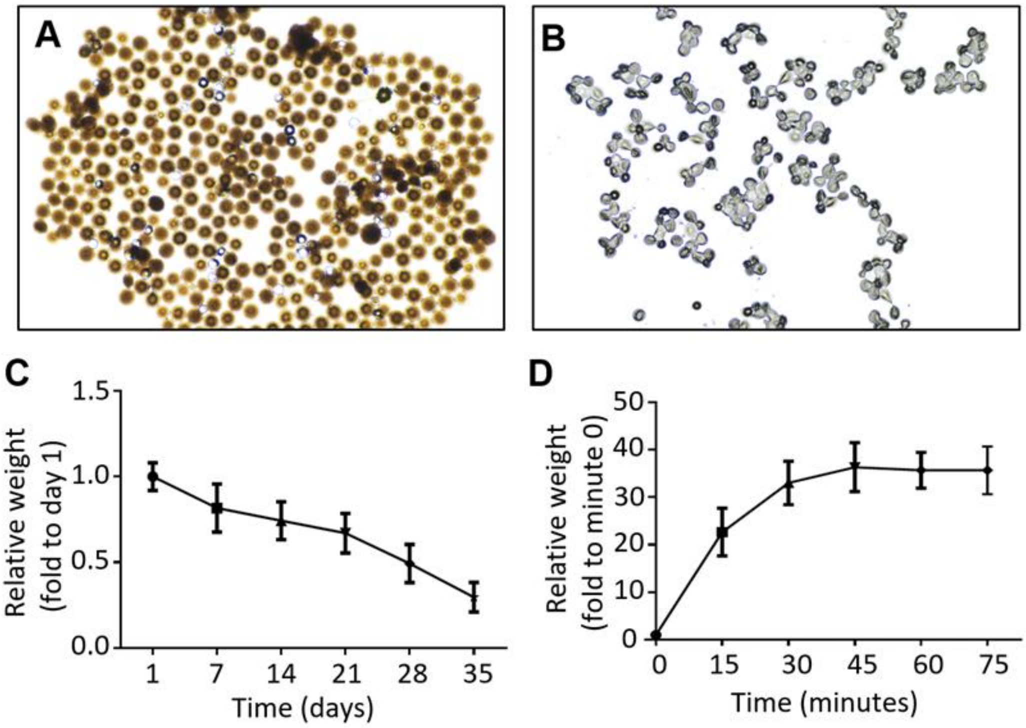

Figure S5. The degradation rate and swelling ratio of hydrogel. A) The image of optical microscope showing the morphological of hydrogel in PBS containing collagenase (1 UmL^{-1}) at 37 °C, and B) the images of degradation at day 35. C) The degradation curve of the hydrogel. D) The swelling curve of the hydrogel.

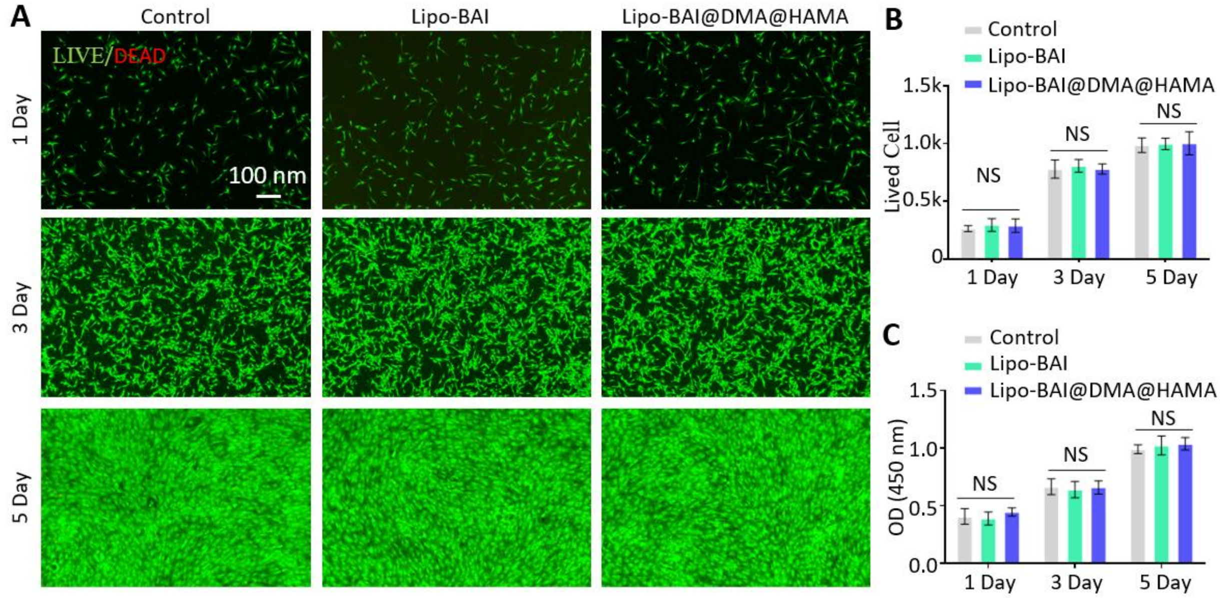

Figure S6. Cytotoxicity of drug-loaded hydrogel. A) The results of live/dead staining about MSC co-cultured with Lipo-BAI, and Lipo-BAI@DMA@HAMA. B) Quantitative analysis of live/dead assay (n = 3). C) The results of CCK-8 assay showed the cytotoxicity of Lipo-BAI, and Lipo-BAI@DMA@HAMA on chondrocyte (n = 5).

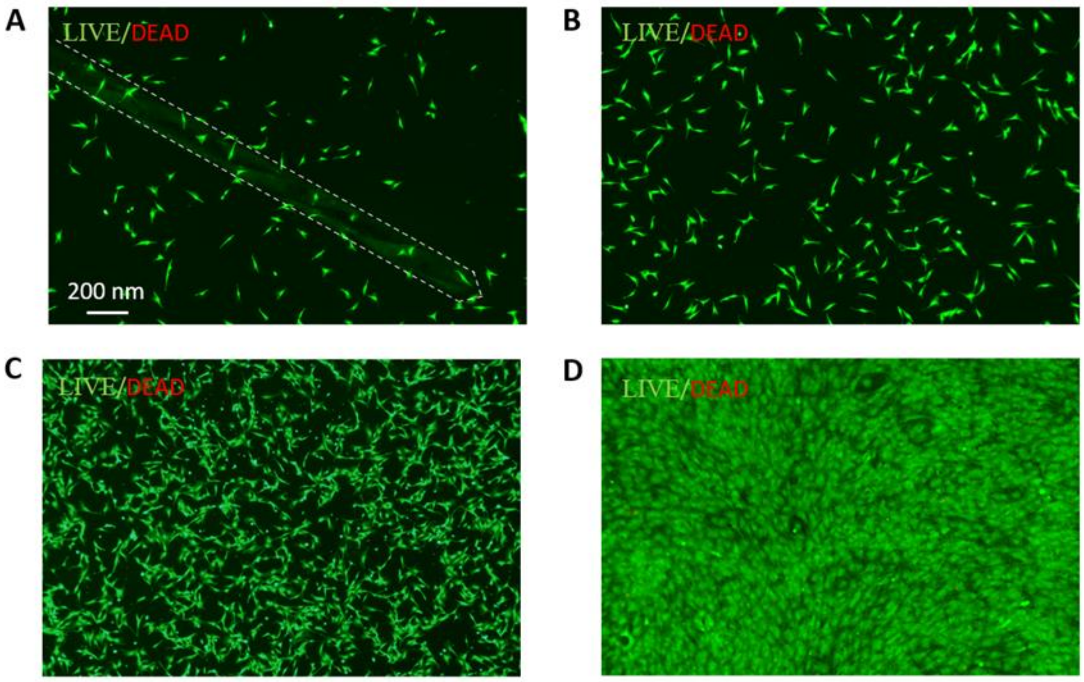

Figure S7. Cytotoxicity of ST-needle. A) The results of live/dead staining about MSC co-cultured with ST-needle for 1 hour (The white dotted line shows the outline of the ST-needle). After the ST-needle were removed from the culture medium, the MSC were cultured for 24 hours (B), 3 days (C), 5 days (D)and then tested with live/dead staining.

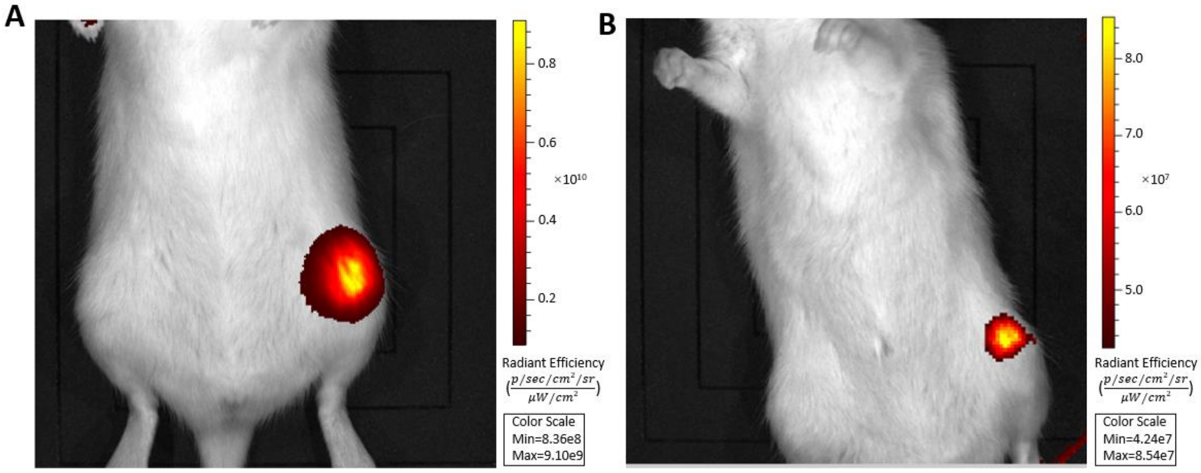

Figure S8. Metabolic rate of free drugs injected into joint cavity. A) Fluorescent intensity right after the drug injection. B) Fluorescence intensity 24 hours after injection.

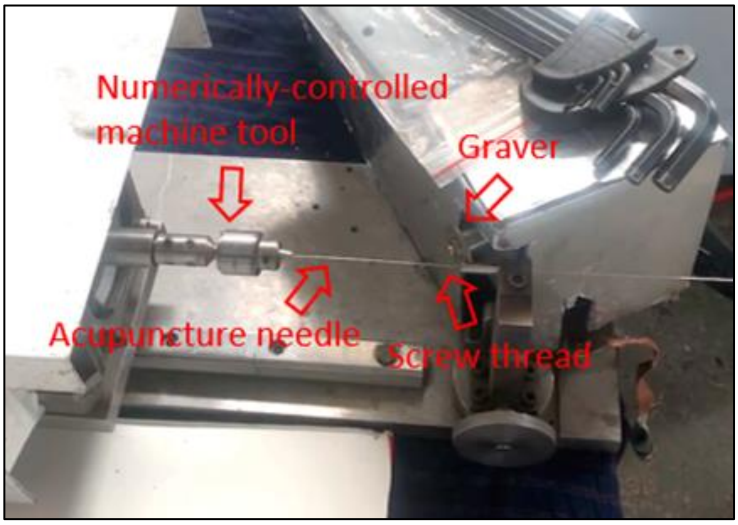

Figure S9. Using numerically-controlled machine tool to produce screw threaded acupuncture needles.

Table S1. Primers used in real-time PCR

Gene	Primer	Sequence
15-LOX-1	Forward	AGCTGTGCAAGACGACTATGAACTG
	Reverse	CGGGACTGAAGAGAGGTAGGGAAG
TGF-β1	Forward	GACCGCAACAACGCAATCTATGAC
	Reverse	CTGGCACTGCTTCCCGAATGTC
GAPDH	Forward	CCTCGTCCCGTAGACAAAATG
	Reverse	TGAGGTCAATGAAGGGGTCGT
